# Trapped air metamaterial concept for ultrasonic sub-wavelength imaging in water

**DOI:** 10.1038/s41598-020-67454-z

**Published:** 2020-06-30

**Authors:** Stefano Laureti, David A. Hutchins, Lorenzo Astolfi, Richard L. Watson, Peter J. Thomas, Pietro Burrascano, Luzhen Nie, Steven Freear, Meisam Askari, Adam T. Clare, Marco Ricci

**Affiliations:** 1 Department of Informatics, Modeling, Electronics and Systems Engineering, University of Calabria, Via Pietro Bucci, 87036 Arcavacata di Rende, CS Italy; 2 School of Engineering, University of Warwick, Coventry, CV4 7AL UK; 3 Department of Engineering, University of Perugia, Polo Scientifico Didattico Di Terni, Via di Pentima 4, 05100 Terni, Italy; 4 School of Electronic and Electrical Engineering, University of Leeds, Leeds, LS2 9JT UK; 5 School of Physics and Astronomy, University of St Andrews, St Andrews, KY16 9SS UK; 6 Department of Mechanical, Material and Manufacturing Engineering, University of Nottingham, University Park, NG7 2RD Nottingham UK

**Keywords:** Materials science, Metamaterials, Acoustics

## Abstract

Acoustic metamaterials constructed from conventional base materials can exhibit exotic phenomena not commonly found in nature, achieved by combining geometrical and resonance effects. However, the use of polymer-based metamaterials that could operate in water is difficult, due to the low acoustic impedance mismatch between water and polymers. Here we introduce the concept of “trapped air” metamaterial, fabricated via vat photopolymerization, which makes ultrasonic sub-wavelength imaging in water using polymeric metamaterials highly effective. This concept is demonstrated for a holey-structured acoustic metamaterial in water at 200–300 kHz, via both finite element modelling and experimental measurements, but it can be extended to other types of metamaterials. The new approach, which outperforms the usual designs of these structures, indicates a way forward for exploiting additive-manufacturing for realising polymer-based acoustic metamaterials in water at ultrasonic frequencies.

## Introduction

The properties of a given material are typically characterized by a number of parameters such as electrical permittivity $\epsilon$ and magnetic permeability $\mu$ for electromagnetic waves, or by the density $\rho$ and bulk modulus $K$ for acoustics. These parameters normally assume positive values for natural materials. In 1968, Veselago^1^ showed theoretically that phenomena such as negative refractive index could be obtained when these parameters are negative. Acoustic metamaterials (AMs) can exhibit exotic properties by using sub-wavelength features (referred as meta-atoms) in the form of resonators and scatterers. These are typically arranged in a periodic way so that they behave like a bulk continuous material but with ‘on-demand’ effective properties^2–16^. For example, Zhang et al.^3^ and Yang et al.^4^ describe materials exhibiting negative effective density $\rho_{eff}$ and bulk modulus $K_{eff} ,$ resulting in a negative acoustic refractive index (${\upeta }_{eff} < 0$).

The manipulation of $\rho_{eff}$ and $K_{eff}$ for a given frequency $\left( f \right)$ and wavelength ($\lambda )$ is of interest for ultrasonic imaging, in applications such as nondestructive evaluation^17–20^ and diagnostic biomedical imaging^21–23^. This arises because of the so-called “diffraction limit”, a consequence of the inability to capture the evanescent field which carries the finer sub-wavelength details of an image. The minimum feature scale $w$ that can be distinguished during an ultrasonic test is given via the relation^1^

1 $$w \cong c/\left( {2 \cdot f} \right) \cong \lambda /2$$

where $c$ is the speed of sound of the medium. AMs can be used to overcome this diffraction limit, because the resolution of the image will be dictated by their sub-wavelength internal structure and the resulting exotic effective properties. For instance, Holey-Structured Acoustic Metamaterials (HSAMs) have been described which produce this enhanced sub-wavelength imaging resolution in air at audible and low ultrasound frequencies. This is achieved by the coupling of evanescent waves via Fabry–Pérot Resonance (FPR) mechanisms^10–12^. Sub-wavelength imaging is very attractive for many imaging methods, one example being near-field optical microscopy. In ultrasound, sub-wavelength imaging could lead to significant improvements. For example, one consequence for biomedical imaging is that lower frequencies could be used with an AM to obtain the same imaging resolution as that from a conventional measurement at higher frequencies, and this would allow an increased penetration into the body.

In their pioneering work^24^, Christensen et al*.* showed that FPRs exist within the holes of a 2D array of square-shaped apertures fabricated within a bulk material. Consider a HSAM having a thickness $h$, through-thickness channels of width $a$ (hole width), and a distance between hole centres, i.e. the lattice constant, $\Lambda .$ Usually $h > \Lambda > a$. The transmission process of acoustic waves through such HSAM is regulated by the resonant mode within each hole, and the transmission coefficient $T^{00}$ is unity when resonances occur within the through-thickness channels, so that

2 $$\lambda_{{FPR_{m} }} = \frac{2 \cdot h}{m} \to \left| {T^{00} } \right| = 1,$$

where $\lambda_{{FPR_{m} }}$ is the wavelength at resonance and $m = 1,2, \ldots ,N$ with $N \in {\mathbb{N}}$.

Equation (2) means that all the acoustic information, including that from evanescent waves, is transferred from one side of the metamaterial to the other whenever the perpendicular component of the incident field wavevector $k_{ \bot }$ is such that $\frac{2\pi }{{k_{ \bot } }} = \lambda_{{FPR_{m} }}$, as the transmission coefficient has a modulus of unity^11^.The resolution is then not dictated by the global material properties but by the lattice constant $\Lambda$, i.e. the final resolution can go well-beyond the limit set by Eq. (1); in addition, $\Lambda /a$ also determines the $\rho_{eff}$ of the HSAM^11,12,25–29^. Examples of imaging in air at resolutions of up to $w$/50 at audible frequencies have been reported^11,12^, where typically the substrate material of the HSAMs has a large acoustic impedance ($Z$) compared to the medium inside the hole, confining the acoustic energy within each channel. For the case of a polymer immersed in water, the difference in acoustic impedance is much less, resulting in an exchange of acoustic energy between the holes. This is likely to lead to a loss in performance, as the effect relies on independence of the channels to preserve the conditions for evanescent wave interaction. It is thus essential that there is a sufficient difference in $Z$ between the bulk material ($Z_{b}$) of the AM and the water-filled holes ($Z_{water}$) within which the acoustic signal is contained. Metallic substrates could be used; however, there is an attractiveness to using polymers as they are readily translated to additive manufacturing technologies, where the use of metallic substrates is limited due to the high aspect ratio $h/a$ of the holes and their relatively-high cost.

Although some work has been reported showing polymer-based HSAMs operating in water^30,31^, the effects of acoustic coupling from the water-filled holes into the polymer substrate would be expected to degrade their performance. As an example, Amireddy et al.^31^ demonstrated operation in water at $f = 250$ kHz, but significant unwanted acoustic energy within the solid was seen, and an amplitude enhancement due to the additional evanescent wave contribution to the image was not observed, as would be expected for efficient operation of an AM^11^. Note that Estrada et al.^13^ reported that conventional polymer substrates would not be expected to be efficient for producing such devices for water-coupled applications.

In this work, we overcome the above-mentioned limitations and demonstrate that polymer-based HSAM structures can in fact be constructed for efficient use in water. This uses a new “trapped air metamaterial” (TAM) concept, where the acoustic impedance mismatch between a polymer and water is strongly enhanced if air is trapped within the bulk material in a particular way. This uses the fact that the acoustic impedance of air ($Z_{air}$) is very low (so that $Z_{air}$ << $Z_{b}$, $Z_{water}$), enhancing acoustic isolation. To gain a quantitative insight into the impedance mismatch problem, consider both nickel ($\rho_{nickel} = 8,900 \,{\text{kg}}\,{\text{m}}^{ - 3}$, $c_{nickel} = 5,600 \,{\text{m}}\,{\text{s}}^{ - 1}$) and a typical polymer ($\rho_{polymer} = 1,400\, {\text{kg}}\,{\text{m}}^{ - 3}$, $c_{polymer} = 1,500 \,{\text{m}}\,{\text{s}}^{ - 1}$), which are the types of materials to be compared in this paper. When used in air at room temperature ($\rho_{air} = 1.2\, {\text{kg}}\,{\text{m}}^{ - 3}$, $c_{air} = 343 \,{\text{m}}\,{\text{s}}^{ - 1}$), the acoustic impedance mismatch of the two materials with respect to that of the air $Z_{air}$ is large, having values of:

3 $$\begin{aligned} & \frac{{Z_{nickel} }}{{Z_{air} }} = 1.2 \times 10^{5} ; \\ & \frac{{Z_{polymer} }}{{Z_{air} }} = 5.1 \times 10^{3} . \\ \end{aligned}$$

This results in efficient confinement of acoustic energy within each single channel in both cases^9–13,32,33^. However, if the same polymer was used with water-filled channels ($\rho_{water} = 1,000\, {\text{kg}}\,{\text{m}}^{ - 3}$, $c_{water} = 1,480\, {\text{m}}\,{\text{s}}^{ - 1} )$, then we obtain

4 $$\frac{{Z_{polymer} }}{{Z_{water} }} = 1.4,$$

so that now energy will pass much more easily from water into the polymer substrate. However, if a layer of air is trapped between the channels and the polymer substrate, we obtain

5 $$\frac{{Z_{water} }}{{Z_{air} }} = 3.6 \times 10^{3} ,$$

i.e. of the same order of magnitude as in Eq. (3), meaning that the novel strategy can be used successfully in water. This can be investigated in more detail via Finite Element Modelling, as showed in the next section. Note that the resultant TAM is expected to outperform the use of metal (nickel) for constructing the metamaterial; this is because:

6 $$\frac{{Z_{nickel} }}{{Z_{water} }} = 33.6 \ll \frac{{Z_{water} }}{{Z_{air} }}.$$

For a last overview of the impedance mismatch issue, the theoretical transmission coefficients values $t_{1 - 2} = 1 - \left[ {\left( {Z_{2} - Z_{1} )/(Z_{2} + Z_{1} } \right)} \right]^{2}$ for the above mentioned combinations are:

7 $$\begin{aligned} & t_{polymer - water} = { }0.97; \\ & t_{nickel - water} = 0.11; \\ & t_{air - water} = 0.0011. \\ \end{aligned}$$

The very low value of $t_{air - water}$ is thus likely to improve the performance of a TAM when compared to the use of simple metal or polymer substrate. Finite element modeling (FEM) simulations and experimental measurements demonstrate both the imaging capabilities of the Trapped Air design and the inability of standard polymer HSAMs to operate well in water. An additional comparison to a nickel structure indicates that the proposed new TAM design is more effective.

### Trapped air metamaterial design

**Figure 1:**
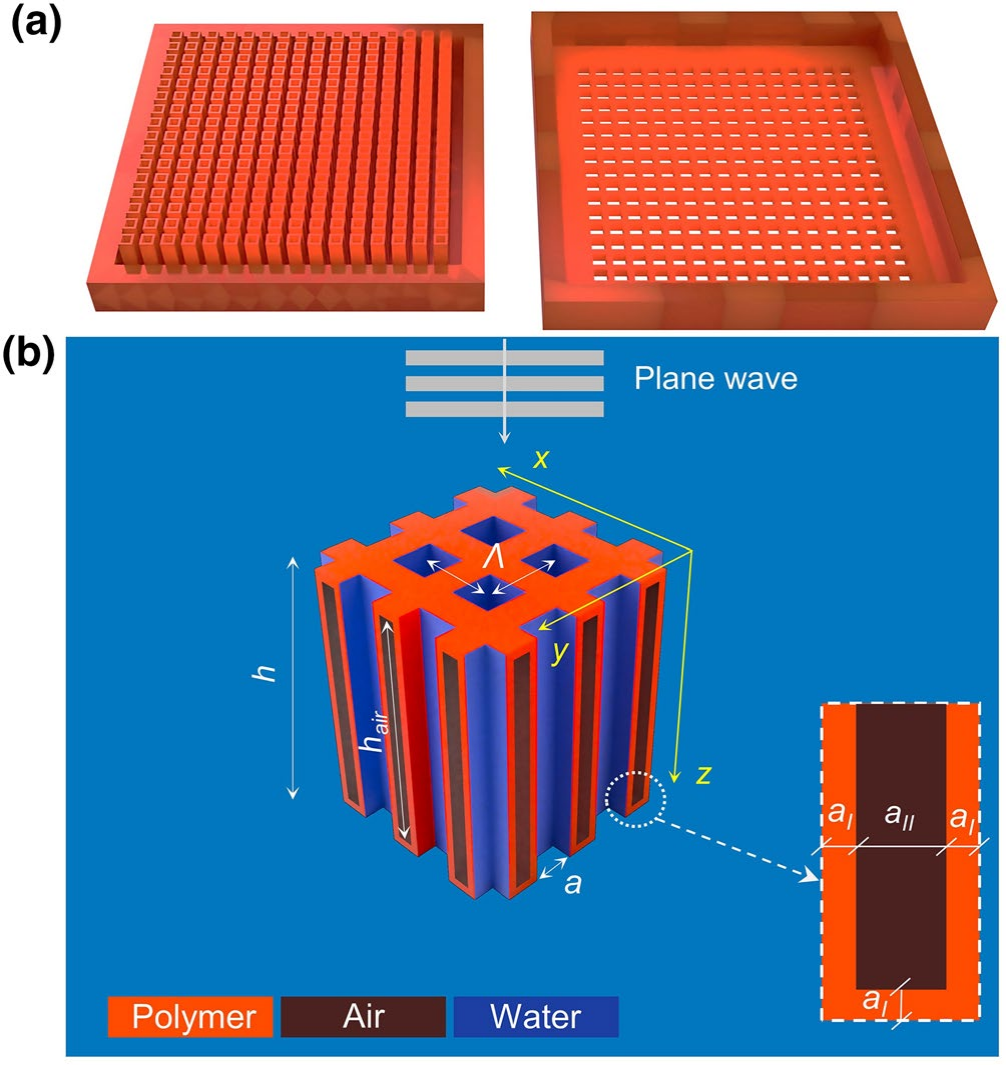
(**a**) The two additively-manufactured layers which, when placed together, form the TAM; (**b**) a sketch representing four unit cells of holes of the TAM. Here $\Lambda$ = 1.2 mm, $a = 0.8$ mm, and $h = 6$ mm, the values used for FEM simulations. Air was trapped within the polymer substrate as shown by the black areas in the zoomed diagram of the embedded air cavity depicted to the right, with $a_{I} =$ 0.1 mm and $a_{II} = 0.2$ mm. Note that the depth of the air cavity is given by $h_{air} = h - 2a_{I} = 5.8 {\text{mm}}$.

The new TAM design was developed to allow a layer of air to completely surround each water-filled channel, in such a way that metamaterials could be constructed in polymer using vat photopolymerization (see "Methods" section). Figure 1a shows the construction of the two layers. This was designed with $\Lambda$ = 1.2 mm, $a = 0.8$ mm, and $h = 6$ mm, producing FPRs in the $f = 100 {-} 300$ kHz range so as to match the available ultrasonic transducer operating bandwidth (see "Methods" section). The bottom layer to the left contains an array of hollow polymer rods, in this case with an air spacing between them. The second layer, shown to the right of Fig. 1a, contains an array of holes to match the channels in each of the hollow rods. This is inverted and placed on top of the first layer and the seal between the two layers made watertight. When immersed in water, the channels would then fill with water, but each would be separated from the other by the air trapped within the sealed device.

A schematic diagram of an assembled TAM is shown in Fig. 1b, with the dimensions used for the FEM simulations also shown. It can be seen how the air layers act to insulate the water-filled channels acoustically from each other.

A TAM was fabricated for experimental testing using the vat photopolymerization process, and a conventional HSAM was also printed using the same polymer for comparison. These were compared to a nickel HSAM fabricated using powder bed fusion techniques. The FEM simulations shown below used values of $a_{I} =0.1$  mm and $a_{II} =0.2$  mm respectively, these values being present in the fabricated polymer and metallic HSAMs. Due to limitations imposed during manufacture of the more complicated TAM structure, (see "Methods" section and Supplementary Information), the walls and air gap had to be thicker in this case ($a_{I} = 0.2 {\text{mm}} \,\,{\text{and}} \,\,a_{II} = 0.6 {\text{mm}})$, and the overall device had fewer holes across its structure (16 × 16 compared to 24 × 24).

**Figure 2:**
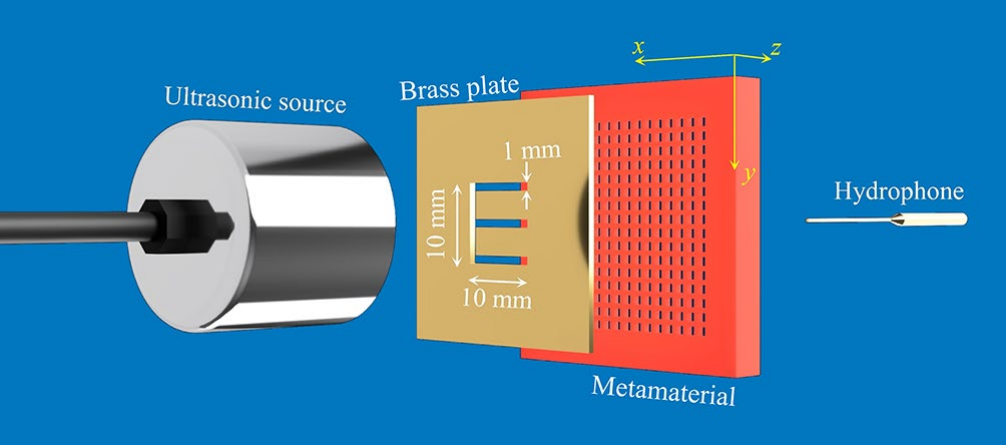
Experimental setup, showing the size and orientation of the “E” aperture, as well as the location of the hydrophone and ultrasonic source with respect to the metamaterial being tested.

The devices were placed in water at room temperature together with an E-shaped aperture machined into a ~ 0.9 mm thick brass plate, as shown in Fig. 2. The arms of the “E” aperture had a width of 1 mm. At the expected simulated FPR of 246 kHz, λ in water is ≈ 6 mm. Hence, for operation at or close to this frequency the aperture was of λ/6 in size, which is beyond the conventional diffraction limit. An ultrasonic piezoelectric transducer was placed at ~ 150 mm from the brass slab and fed with a frequency modulated “chirp” signal over the 100–400 kHz frequency range. A miniature hydrophone (Precision Acoustics) of 0.2 mm active diameter collected the ultrasonic pressure field transmitted through the metamaterial at various locations across the *x*–*y* plane.

The received signals were processed with Chirp Z-Transform algorithm^33^ and images obtained for comparison to FEM predictions.

## Results

### Finite element simulations

**Figure 3:**
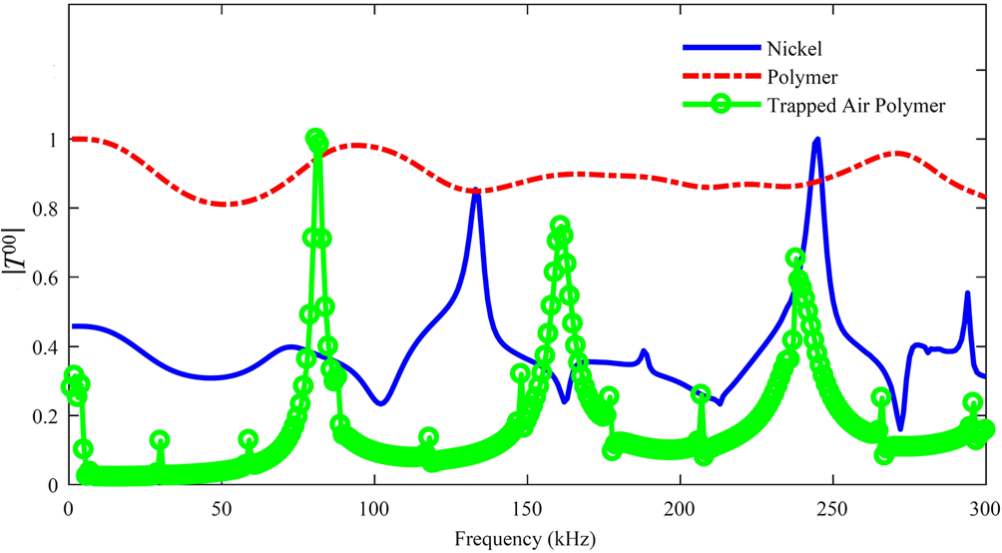
2-D FEM simulations showing absolute values of the total pressure field for polymer and nickel HSAMs, and a trapped air polymer TAM design.

A 2-D FEM was built to obtain the transmission coefficient $\left| {T^{00} } \right|$ of three designs — a TAM in polymer containing trapped air, and HSAMs with simply holes within either a polymer or nickel substrate, each having the same values of $\Lambda$, $a$ and $h$ as shown in Fig. 1b. A plane wave acoustic field propagating along the *z*-axis direction was employed in the modelling together with a perfectly- matched layer domain. Predictions for $\left| {T^{00} } \right|$ were obtained over the $f = 0 {-} 300$ kHz range and are plotted in Fig. 3. The reader is referred to "Methods" section for more details of the FEM model. It is clear that both the new trapped air design and its bulky nickel counterpart lead to the successful establishment of FPRs, whilst a flattened behaviour is obtained for a standard polymer design. This indicates that, without this trapped air acoustic isolation, a severe disruption to the operation of the polymer metamaterial is likely to occur—this is in agreement with the predictions of Eqs. (3)–(5). Moreover, it can be noted that more resonant peaks are seen for the trapped air design with respect to its metal counterpart and to the theoretical prediction of Eq. (2). Thus, the TAM is likely to behave as a medium with an effective speed of sound $c_{eff - TAM} = 972 \,{\text{m}}\,{\text{s}}^{ - 1}$, being estimated from substitution of the value of 81 kHz in Eq. (2) for the first observed FPR mode (*m* = 1).

**Figure 4:**
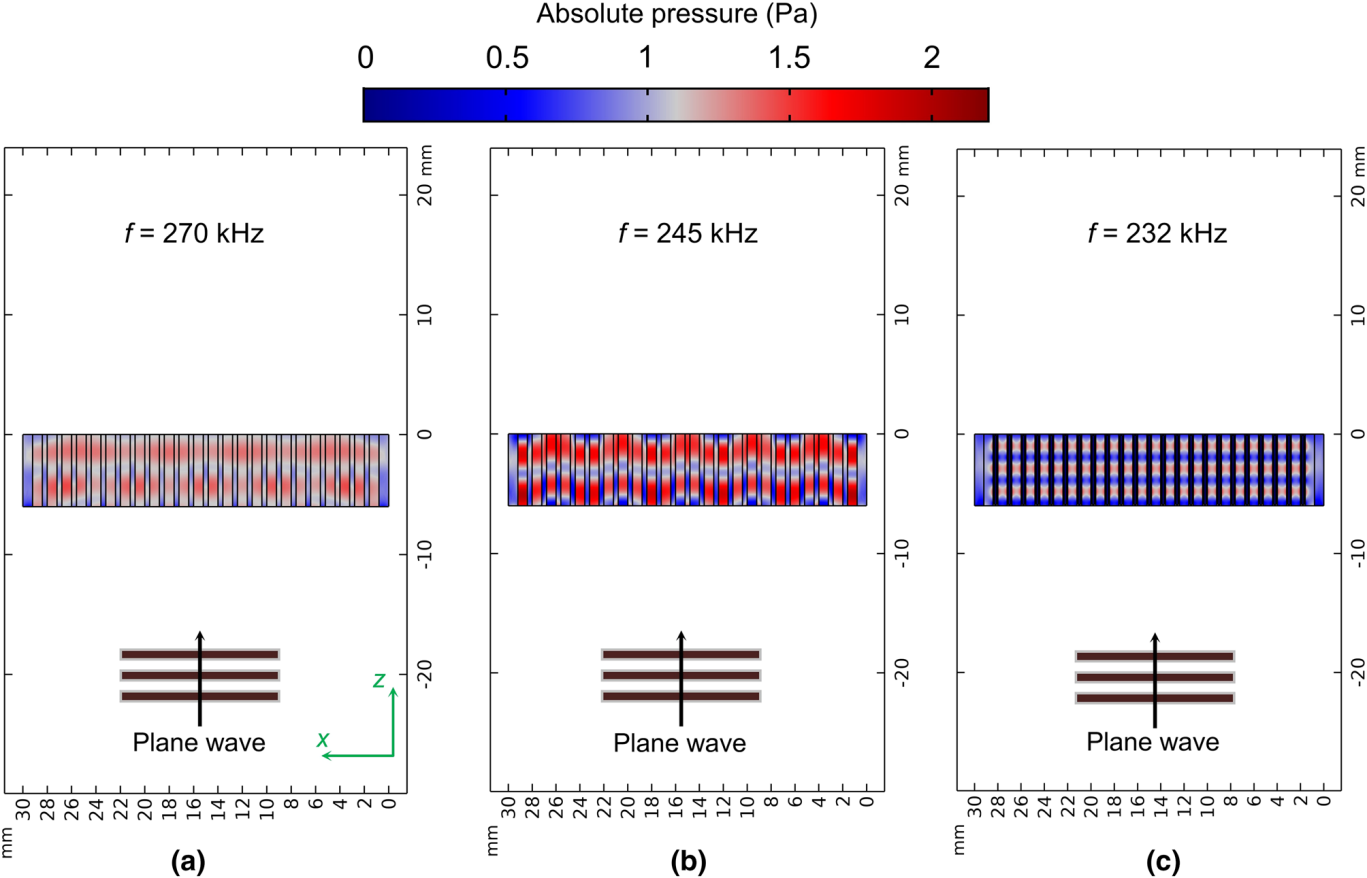
2-D FEM simulations showing absolute values of the total pressure field for (**a**) polymer HSAM, (**b**) nickel HSAM and (**c**) TAM designs.

More insight into the impedance mismatch problem, and thus about the validity of the proposed trapped air design and the establishment of FPR within the holes, is found if $x - z$ sections of the structures are investigated. Figure 4a–c shows the absolute value of the pressure field at peak frequencies for the two HSAMs and the TAM respectively. While FPRs are well-established and isolated from each other in the TAM, the same it is not true for the polymer substrate HSAM, where FPRs are not well-established. This is due to widespread exchange of acoustic energy between individual channels. Finally note that the TAM design show more distinct standing waves/FPRs than those of a nickel HSAM, which agrees with the estimated value of $c_{eff - TAM}$.

### Sub-wavelength imaging

**Figure 5:**
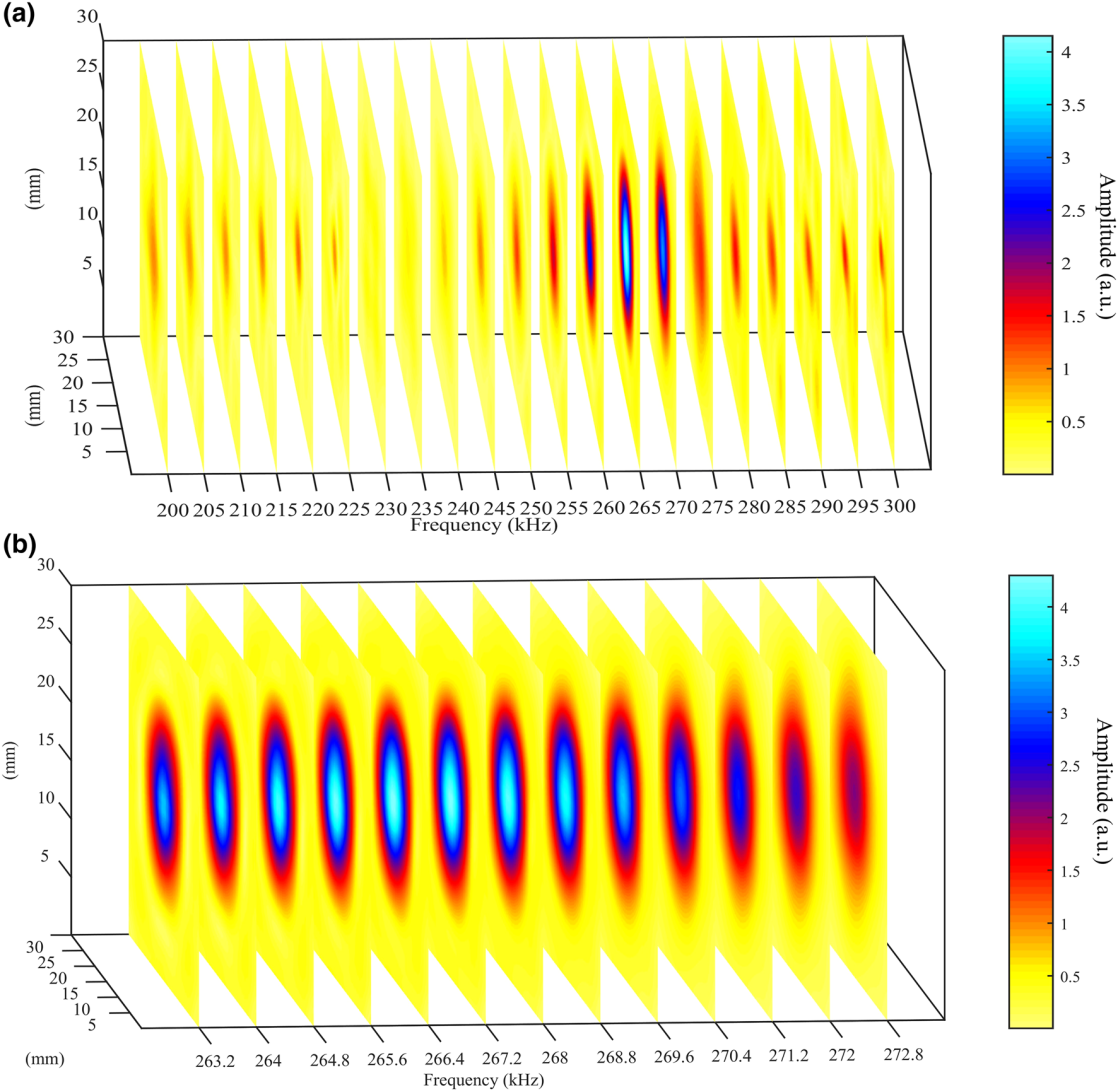
(**a**) A series of $x {-} y$ planes at different frequencies for the polymer HSAM obtained from 3-D FEM simulations. “E” details are completely lost at any frequencies in the 200–300 kHz range; (**b**) details for frequencies around the maximum transmission frequencies values.

**Figure 6:**
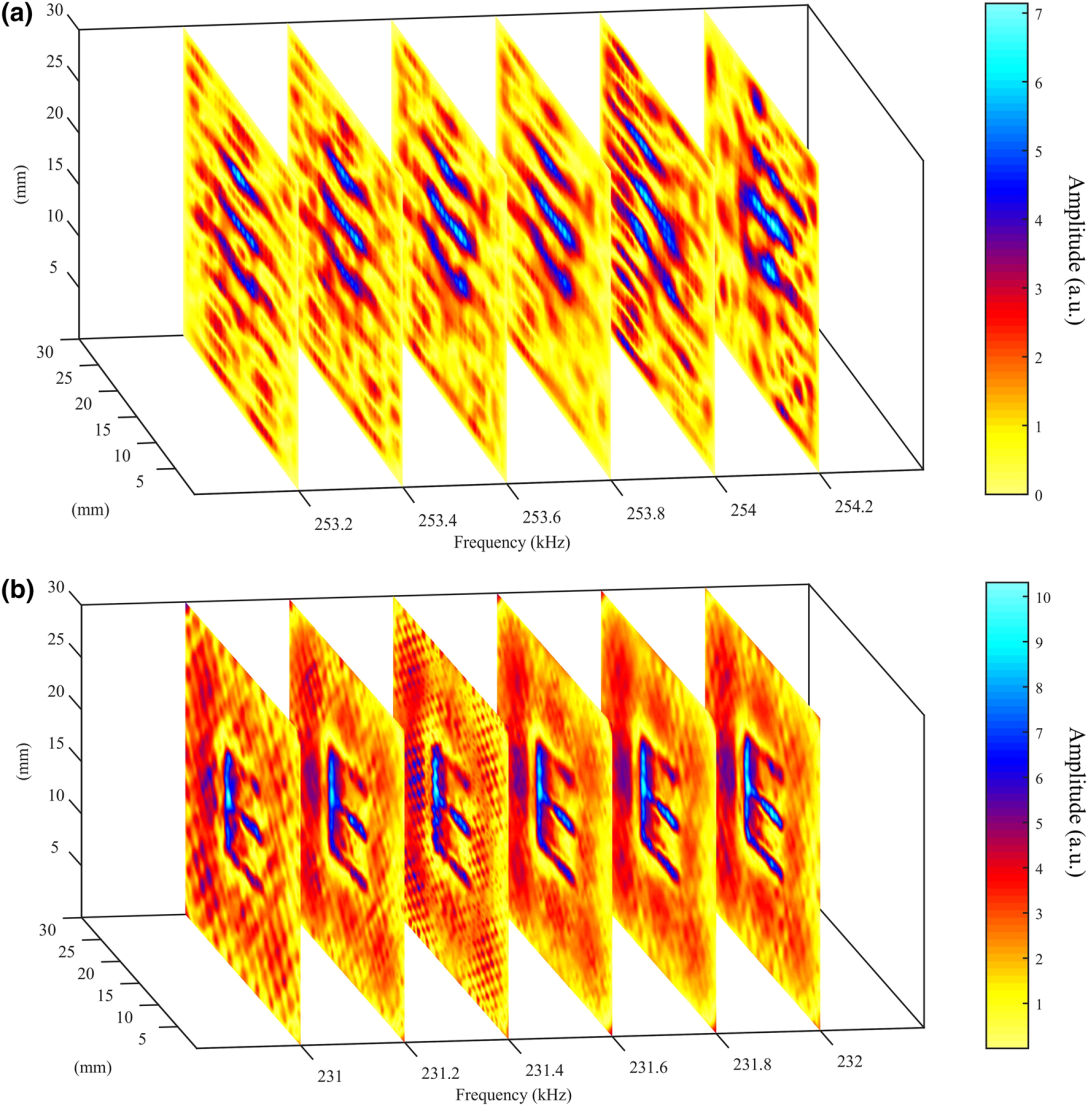
A series of $x {-} y$ planes at different frequencies for (**a**) the nickel and (**b**) the TAM design for frequencies around the resonance peak (obtained from 3-D FEM simulations).

3-D FEM simulations were conducted to check whether sub-wavelength details could be resolved using the three designs, see Figs. 5 and 6. Figure 5 shows a series of two-dimensional sections of the total pressure field magnitude across $x {-} y$ planes for the polymer HSAM design at a fixed location but at different frequencies. In Fig. 5a a wide frequency range is analyzed: sub-wavelength details are lost over the whole range due to the global resonance of the whole channeled structure, but an enhanced transmission occurs around the predicted resonance frequency values. By zooming around the peak, see Fig. 5b, it is also confirmed that sub-wavelength details cannot be appreciated at frequencies where enhanced transmission occur.

Conversely, sub-wavelength imaging capabilities are exhibited at frequencies around a resonance by both nickel HSAM and polymer TAM designs, see Fig. 6a,b respectively. In particular, the TAM shows much better results than the nickel one as all the “E” edges, including the vertical one, are well-imaged.

### Experimental results

**Figure 7:**
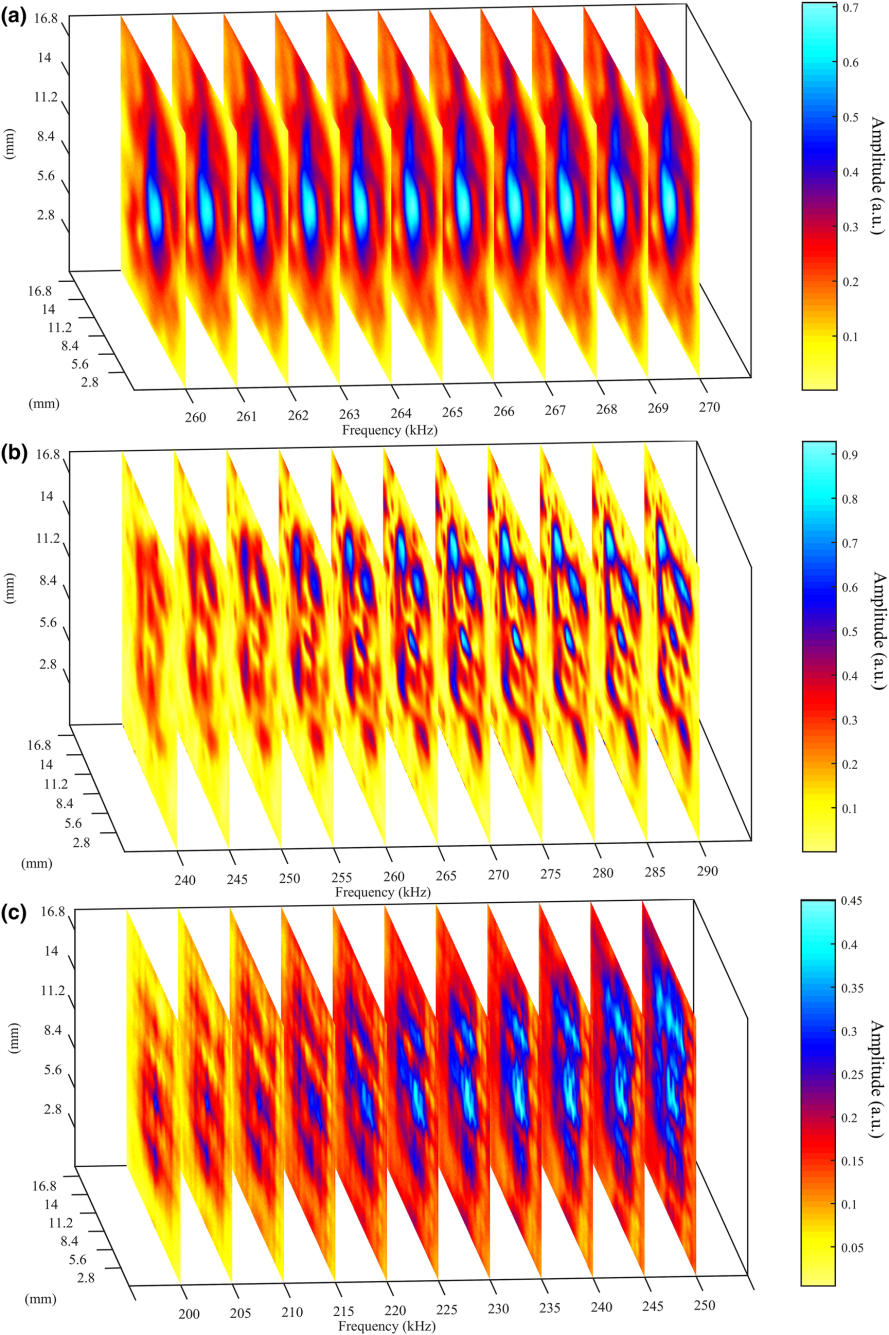
Experimental data showing a series of $x {-} y$ planes at different frequencies obtained by imaging the “E-shaped” sub-wavelength aperture with the (**a**) polymer, (**b**) nickel HSAMs, and (**c**) the TAM.

Experiments were performed in a water tank for all three designs, and the experimental images that were obtained are shown in Fig. 7a–c. Despite the larger resultant hole size and separation in the TAM, the results were excellent, showing the TAM design to be very effective compared to HSAM designs in either polymer or metal. The “E” details are not imaged by the polymer HSAM (Fig. 7a), as expected from the simulations of Fig. 5 and the impedance mismatch prediction of Eq. (4). Conversely, the finer details of the “E” shaped aperture are seen at frequencies close to the FPR frequency values for both the nickel HSAM (Fig. 7b), and TAM (Fig. 7c) designs. Good results are obtained for the TAM, noting that a shift toward the lower frequencies for the best image was obtained experimentally due to its slightly greater thickness ($h$ ~ 6.4 mm, see "Methods" section and Supplementary Information).

The earlier FEM simulations of Fig. 6 predicted that the TAM would outperform the metal HSAM in imaging performance for the same basic unit cell parameters. It should be noted that the HSAMs were simulated and fabricated with a 24 × 24 matrix of holes, based on a unit cell with solid walls (0.4 mm) between each 0.8 mm hole, but that the TAM measured experimentally had a 16 × 16 matrix over the same area. This is because the unit cell spacing had to be increased to allow the polymer walls trapping the air between each hole to be structurally sound. Despite this, the TAM still outperformed the two HSAMs experimentally. Note also that it is the dimensions of the water-filled holes that predominantly control the resonances and frequency response, and so it was essential to retain these dimensions (*a* and *h*) across all samples (both HSAMs and the TAM) for the comparison. The reduction in the density of the holes in a given area of the TAM would have adversely affected both the imaging resolution and the *ρ*_*eff*_ (which is a function of *Λ*/*a*^11,12,25–29^); even so, the TAM was still the best performing structure in our studies.

**Figure 8:**
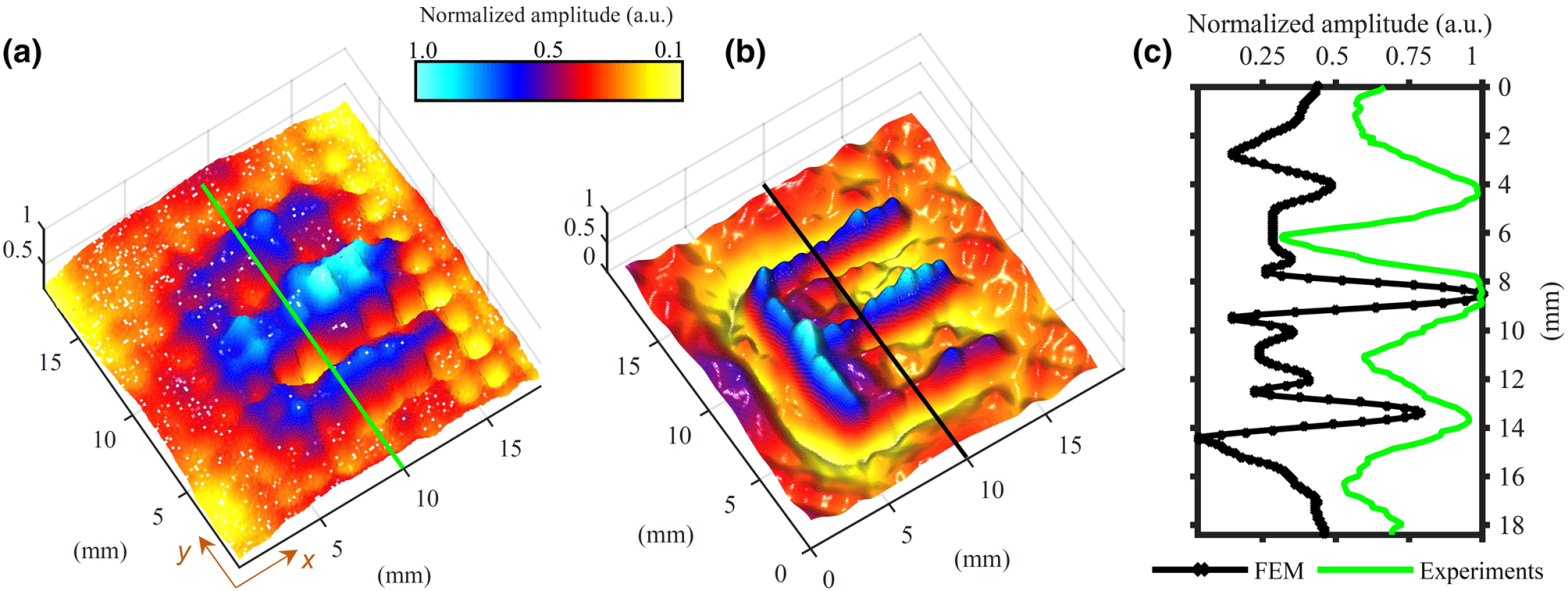
Comparison of (**a**) experimental results in a water tank and (**b**) FEM simulations for the TAM; (**c**) a comparison plot of the normalized pressure amplitude at *x* = 10 mm, corresponding to the green and black lines in (**a**) and (**b**) respectively.

Figure 8a shows the best image experimentally-obtained using the TAM, together with the results obtained from the 3-D FEM simulation (Fig. 8b), and a cross-section of the amplitude obtained at $x = 10$ mm for both the experiment and the simulations (Fig. 8c). A good agreement between the two is found, as the “E” profile is well-reconstructed. This further demonstrates the capability and potential of the trapped air design strategy. Note that the greater separation of the holes of the realised TAM device with respect to the FEM design results in a lower spatial resolution (see Fig. 8c). In fact, by considering the TAM as a periodic structure along the surface plane, $\pi /\Lambda$ determines the limit of the Brillouin zone, so the smaller is $\Lambda$, the larger is the Brillouin zone^11,12,25–29^ . However, it is still evident that the TAM is still an excellent structure for imaging purposes.

## Conclusions

It has been shown that the use of trapped air within an acoustic metamaterial fabricated from polymers in a standard additive manufacturing process can enable sub-wavelength imaging in water. The performance of the resulting TAMs has been modelled and shown to be similar to that obtained experimentally at ultrasonic frequencies in water. As expected, the air layers have greatly reduced acoustic cross-coupling from the water-filled channels into the polymer substrate, thus enabling a new class of acoustic metamaterial to be considered for ultrasonic sub-wavelength imaging, sound shielding/extraordinary transmission, and in other acoustic metamaterial designs where high-impedance mismatches between channels are strongly desired^3,4,33^.

## Methods

A detailed description of the materials and methods employed in the present research is reported below.

### 2-D finite element modelling simulations

**Figure 9:**
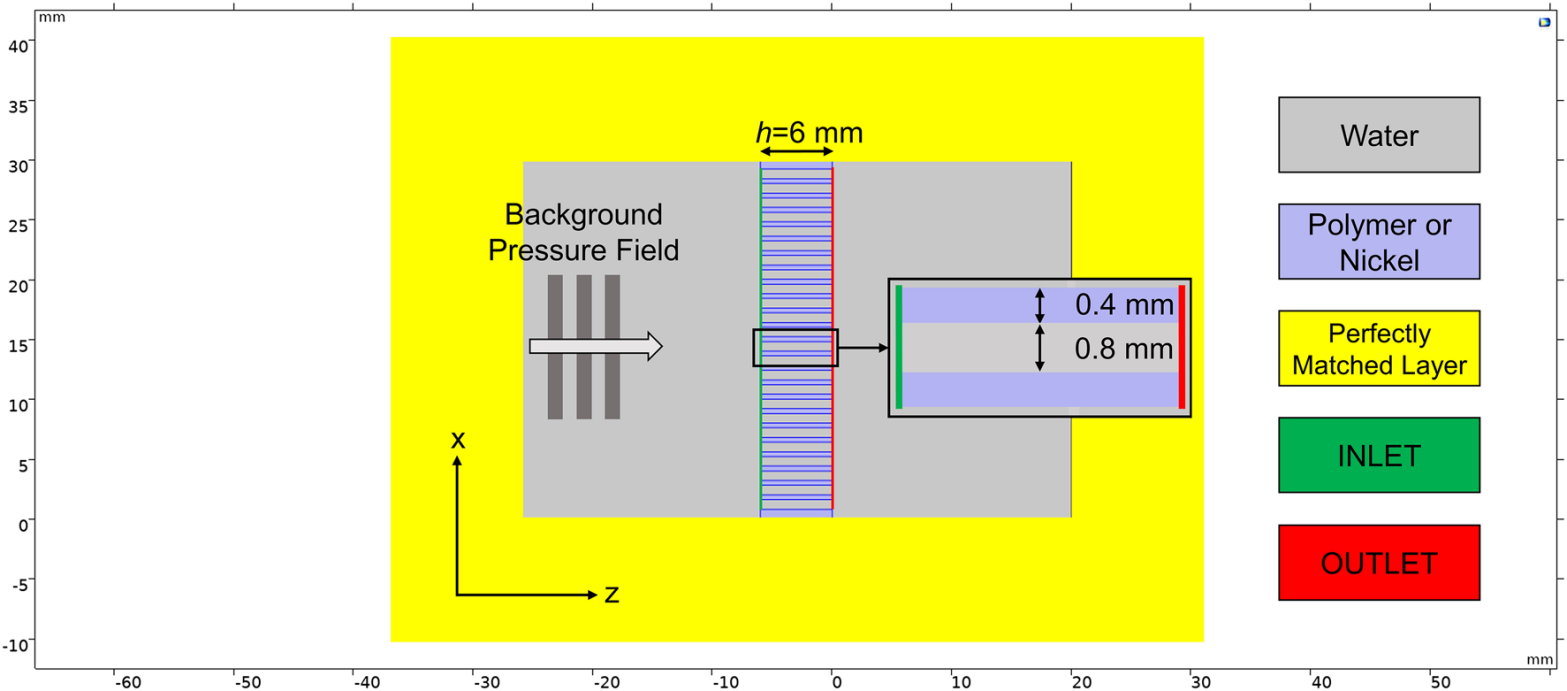
2-D FEM simulation geometry of a polymer/nickel HSAM with 24 holes.

A 2-D pressure acoustics frequency domain finite element model was realised in COMSOL Multiphysics to characterise the frequency behaviour of the tested HSAMs. Two different geometric arrangements were tested: one for simulating either polymer or nickel HSAMs, while the latter employed a volume of trapped air enclosed in a polymer shell. The geometry employed for the polymer and nickel cases is illustrated in Fig. 9.

**Figure 10:**
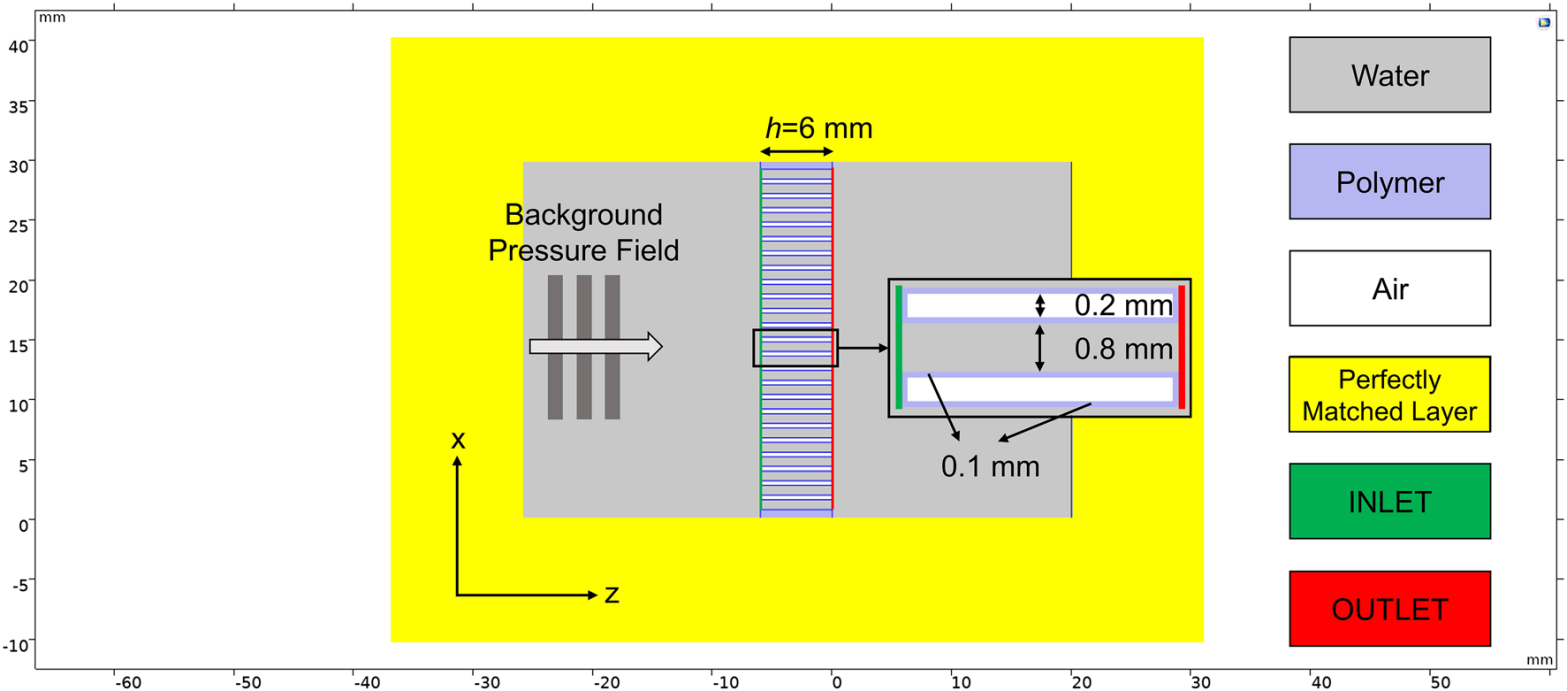
2-D FEM simulation geometry of TAM containing 24 holes.

A control volume of 20 mm was used both before and after the metamaterial in the *z*-direction, and the whole geometry was surrounded by a 10 mm perfectly matched layer (PML) to simulate an infinite water tank, i.e. anechoic condition. The geometry was meshed so to have at least 10 elements to represent each channel width. A 1 Pa (peak-to-peak) plane wave travelling along the *z* direction was incident onto the AM at different frequencies in the 100–300 kHz range (at 100 Hz increments). The transmission coefficient $\left| {T^{00} } \right|$ of each structure was obtained as the ratio of the outlet pressure energy to that at the inlet. A different geometry was employed to demonstrate the effect of the trapped air inside the polymer, represented in white in Fig. 10.

## 3D finite element modeling simulations for sub-wavelength imaging

**Figure 11:**
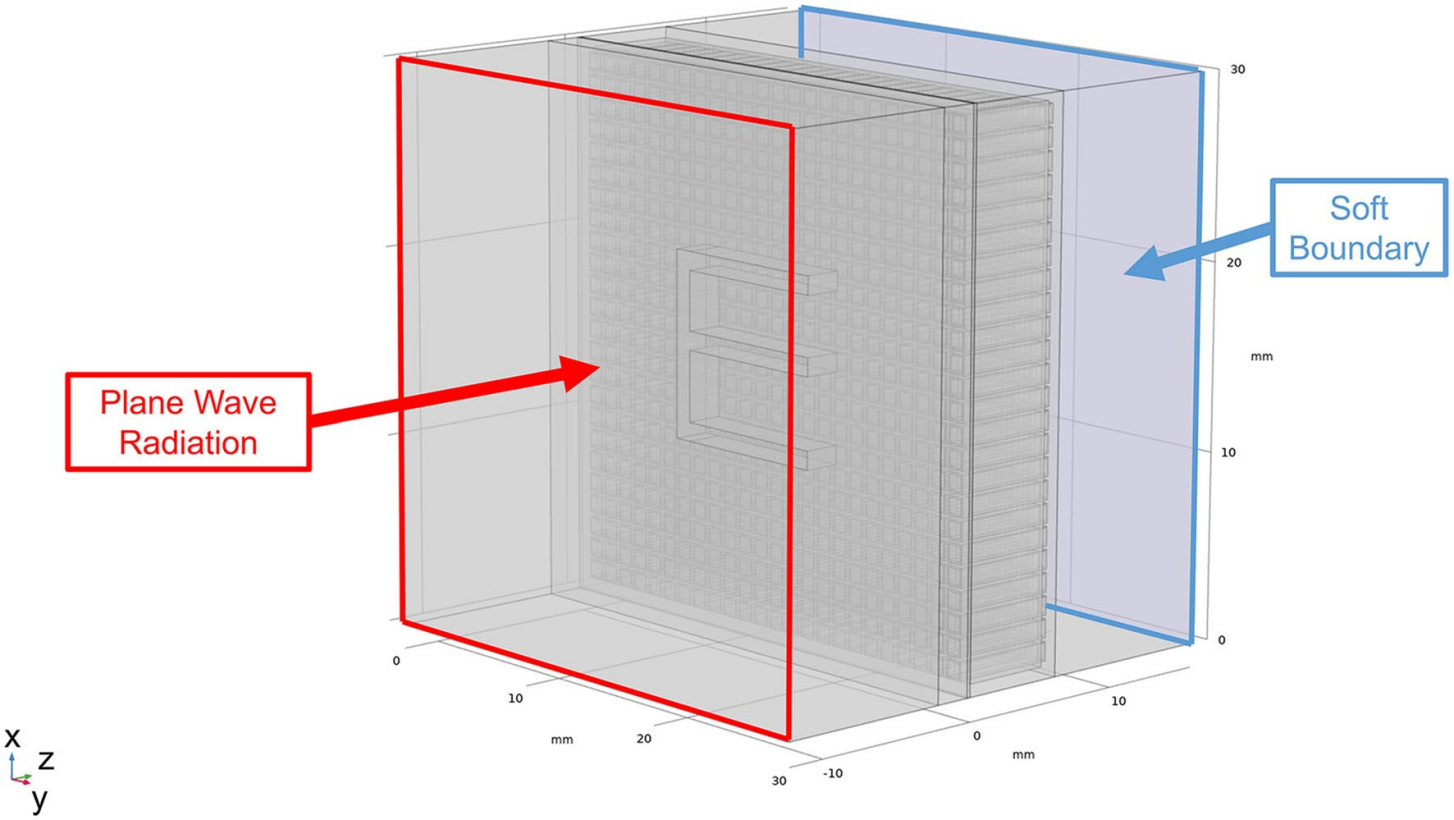
3-D FEM simulations geometry for the study of sub-wavelength imaging.

The 3D model consisted of a plane wave travelling through an “E” shaped aperture having sub-wavelength thickness, carved out of a 1 mm thick brass plate. A plane wave radiation condition was applied to the first boundary in *z* direction and a soft boundary condition was applied to the last boundary in *z* direction to avoid reflections. Data were analysed by imaging the pressure field amplitude at 0.1 mm from the HSAMs outlet, see Fig. 11.

### HSAMs additive manufacturing

The TAM was manufactured using vat polymerisation containing a 16 × 16 array of holes with $a = 0.8$ mm, $h = 6$ mm, $\Lambda = 1.8$ mm, $a_{I} = 0.2$ mm and $a_{II} = 0.6$ mm (see Fig. 1a for a diagram of dimensions). These differ from the FEM intend due to the manufacturing tolerances on wall thickness ($a_{I}$) and air gap ($a_{II}$) to allow reliable production. Vat polymerisation utilised Daylight precision hard white resin (25 µm layer height) and a Liquid Crystal Precision 1.5 3-D printer both supplied by the Photocentric Group. All additive manufacturing preparation required the controlled use of support material to prevent any support being applied to the holes which would occlude them. Vat polymerisation was also used for the polymer HSAM. Metallic HSAM was produced through powder bed fusion of Inconel 625 (a Nickel based alloy) with an array of 24 × 24 holes with $a = 0.8$ mm, $h = 6$ mm, and $\Lambda = 1.2$ mm. This used a Renishaw AM250 SLM Powder Bed Fusion printer with a laser power of 200 W and a laser spot size of 150 µm. This conventional design was also manufactured for comparison in vat polymerisation to allow material comparison without trapped air gaps. This used the same materials and equipment settings as those used for the TAM.

### Experimental setup

The experiments were conducted in a 400 mm long, 350 mm wide and 200 mm deep volume of water enclosed in a custom-made acrylic tank. The input voltage signal was generated by a NI PXI-5421 Arbitrary Waveform Generator, amplified by a NCA1000-2E amplifier and emitted by a custom made 25.4 mm diameter piezocomposite transducer. An ultrasonic chirp signal of 50 µs duration and with frequencies sweeping from 100 to 400 kHz was used. The radiated ultrasonic signal passed through a ⁓ 0.9 mm thick plate containing the sub-wavelength aperture in the form of the letter “E”. The through-transmitted energy was then collected by the tested metamaterials (one at a time), and transferred to its far side to be then acquired at a distance of ⁓ 0.15 mm from the outlet surface using a 0.2 mm diameter Precision Acoustics needle hydrophone. Signals were then captured using a National Instruments PXI-5122 14 Bit Digitizer sampling at a rate of 100 MS·s^−1^. Both the Waveform Generator and the Digitizer were enclosed in a NI PXI-1042Q chassis. A grid of 131 × 131 measurement points was acquired by means of a 3D motorized stage with steps of 0.14 mm for an area of 18.34 × 18.34 mm^2^. In order to approximate a flat far field wave front, the distance between source and hydrophone was chosen to be 150 mm. Note that the setup employed is similar to that described in^34^.

For each scanned point, the DC component was removed from the time domain waveform and the region of interest, selected via a rectangular time window, was converted into frequency domain data via a Chirp Z-Transform algorithm^33^.

## Supplementary information

Supplementary information

## Data Availability

The datasets generated during and/or analysed during the current study are available from the corresponding author on reasonable request.

## Code availability

The FEM simulations were performed using COMSOL 5.4 (licence no. 7077568). The code is available from (lorenzo.astolfi@warwick.ac.uk) on reasonable request.
