# Supplementary material for: Trapped air metamaterial concept for ultrasonic sub-wavelength imaging in water
Source: Sci Rep. 2020 Jun 30;10:10601. doi: 10.1038/s41598-020-67454-z (PMC7326974; doi:10.1038/s41598-020-67454-z)
Supplement: Supplementary file 1 — Supplementary information [file 41598_2020_67454_MOESM1_ESM.docx]

**Supplementary Information**

**Trapped Air metamaterial concept for ultrasonic sub-wavelength imaging in water**

Stefano Laureti, David A. Hutchins, Lorenzo Astolfi, Richard L. Watson, Peter J. Thomas, Pietro Burrascano, Luzhen Nie, Steven Freear, Meisam Askari, Adam T. Clare and Marco Ricci

**Experimental Results**

Additional experiments (see Method) were conducted to establish imaging capabilities without the metamaterial. Figure 1 shows a series of images at different frequencies for the E”-shaped aperture with sub-wavelength dimensions, whereby the hydrophone was placed at 0.1 mm from the slab’s outlet without any HSAMs in between. It is noticed that finer details of the “E” are not imaged, as would be expected for a sub-wavelength (1 mm wide) aperture and a detector of finite diameter (0.2 mm).

**
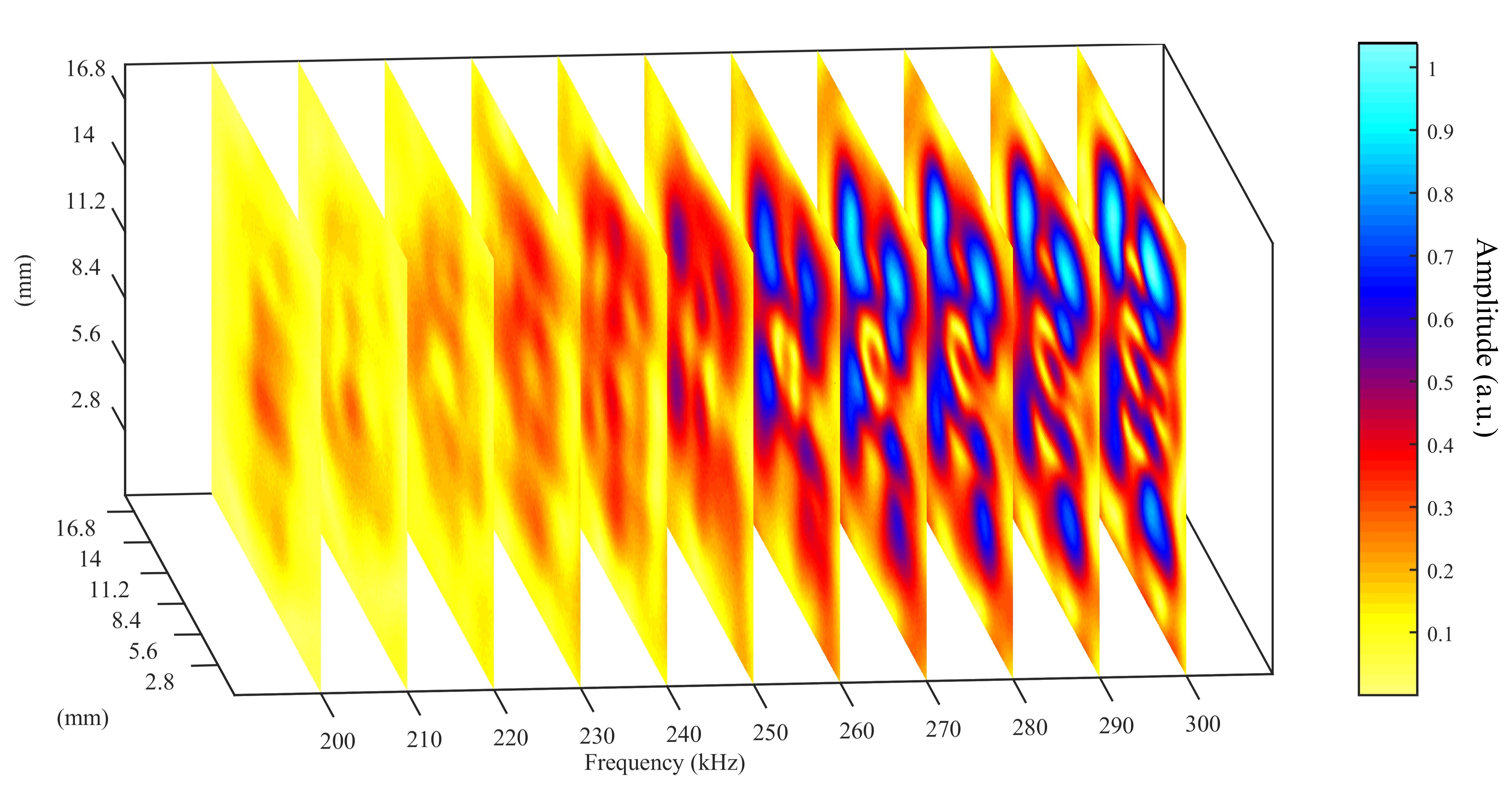
**Figure 1: Experimental results for imaging the “E-shaped” sub-wavelength aperture at a series of $x-y$ planes at different frequencies without a metamaterial being present. The hydrophone was positioned as close as possible to the aperture.

Polymer, nickel, and TAM HSAMs were fabricated by additive manufacturing (see Method) and placed between the “E” aperture and the hydrophone, and photographs are shown in Fig.2 (a-c). Note that there was a 0.1 mm gap between the aperture and the HSAMs.


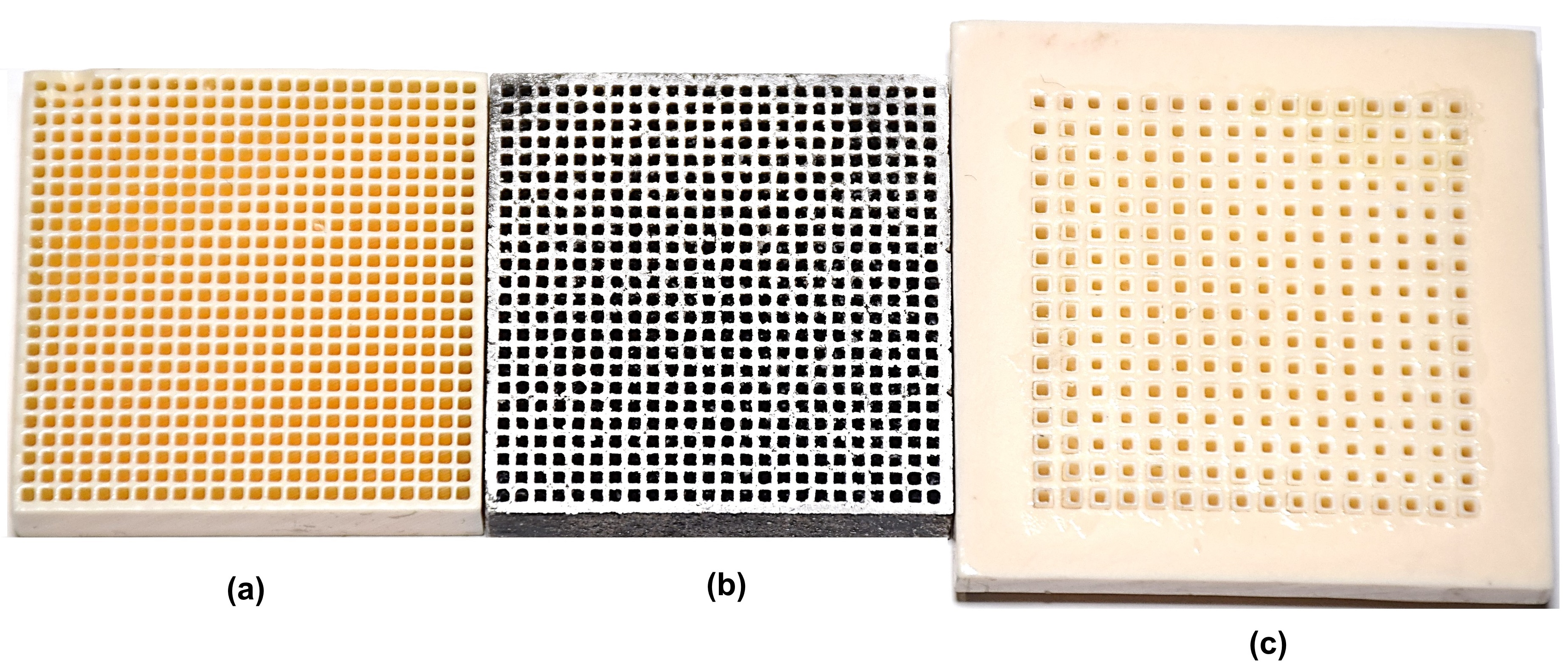
Figure 2: Photographs of the additively-manufactured (a) polymer, (b) nickel, and (c) TAM HSAMs.
